# LincR-PPP2R5C deficiency enhancing the fungicidal activity of neutrophils in pulmonary cryptococcosis is linked to the upregulation of IL-4

**DOI:** 10.1128/mbio.02130-24

**Published:** 2024-09-17

**Authors:** Chen Yang, Ying Gong, Shan Liu, Chunan Sun, Ting Wang, Xin Chen, Wei Liu, Xia Zhang, Yonglin Yang, Mingshun Zhang

**Affiliations:** 1 Department of Immunology, National Vaccine Innovation Platform, School of Basic Medical Sciences, Nanjing Medical University, Nanjing, China; 2 NHC Key Laboratory of Antibody Technique, Jiangsu Key Laboratory of Pathogen Biology, Department of Immunology, Nanjing Medical University, Nanjing, China; 3 Department of Respiratory and Critical Care Medicine, The First Affiliated Hospital of Nanjing Medical University, Nanjing, China; 4 Department of Infectious Diseases, The Affiliated Taizhou People’s Hospital of Nanjing Medical University, Taizhou School of Clinical Medicine, Nanjing Medical University, Nanjing, China; Institut Pasteur, Paris, France

**Keywords:** long non-coding RNA, *Cryptococcus neoformans*, pulmonary cryptococcosis, IL-4, macrophage, neutrophil

## Abstract

Pulmonary cryptococcosis is a common complication in immunocompromised patients. In a mouse model of pulmonary cryptococcosis, *Cryptococcus neoformans* induces a type 2 immune response that is detrimental to host protection. Long non-coding RNAs (lncRNAs) have emerged as key players in the pathogenesis of infectious diseases. However, the roles and mechanisms of lncRNAs in fungal infection are largely elusive. In the present study, we aimed to explore the roles of LincR-PPP2R5C in pulmonary cryptococcosis. We observed an increase in the level of LincR-PPP2R5C in the lung tissues of C57BL/6J mice after tracheal infection with *C. neoformans*. Subsequently, we intratracheally infected LincR-PPP2R5C knockout (KO) mice and wild-type mice with *C. neoformans*. LincR-PPP2R5C deficiency mitigates *C. neoformans* infection, which can be demonstrated by extending survival time and decreasing fungal burden in the lung. In the lung tissues of infected LincR-PPP2R5C KO mice, there was a notable increase in the levels of type 2 cytokines [interleukin (IL)-4 and IL-5] and an increase in the number of neutrophils in both the lung tissue and bronchoalveolar lavage fluid. Mechanistically, the lack of LincR-PPP2R5C results in increased protein phosphatase 2A phosphorylation, thereby enhancing the fungicidal activity of neutrophils against *Cryptococcus neoformans*, with IL-4 playing a synergistic role in this process. Overall, LincR-PPP2R5C deficiency mitigated pulmonary cryptococcosis by increasing the fungicidal activity of neutrophils, which was associated with increased IL-4 levels. Our study presented specific evidence of the role of host-derived lncRNAs in the regulation of *C. neoformans* infection.

**IMPORTANCE:**

Pulmonary cryptococcosis is a human fungal disease caused by *Cryptococcus neoformans*, which is common not only in immunocompromised individuals but also in patients with normal immune function. Therefore, studying the control mechanisms of pulmonary cryptococcosis is highly important. Here, we demonstrated that the deletion of LincR-PPP2R5C leads to increased killing of *C. neoformans* by neutrophils, thereby reducing pulmonary cryptococcal infection. These findings will greatly enhance our understanding of the mechanisms by which lncRNAs regulate the pathogenesis of *C. neoformans*, facilitating the use of lncRNAs in pulmonary cryptococcosis therapy.

## INTRODUCTION

Invasive fungal pathogens cause approximately 300 million infections and 1.5 million deaths each year (1). *Cryptococcus neoformans* is an opportunistic pathogenic fungus that can be inhaled into the lungs through the respiratory tract. Pulmonary cryptococcosis is an important opportunistic invasive fungal disease in immunocompromised patients, but it is also increasingly common in patients with normal immune function (2, 3). In 2022, the World Health Organization released the list of fungal priority pathogens for the first time in history, and *C. neoformans* ranked first in the list. More efforts are required to elucidate the pathogenesis of *C. neoformans* infection.

It has been historically recognized that both macrophages and neutrophils can kill *C. neoformans* and jointly participate in the first-line defense against *C. neoformans* during the course of infection (4). Neutrophils, one of the most abundant types of immune cells in the blood, are critical for the killing and regulation of *C. neoformans* during initial infection and show greater antifungal effects than monocytes and macrophages (5, 6). After infection, circulating neutrophils migrate to the site of infection to help with fungal clearance. They can kill *C. neoformans* both intracellularly and extracellularly through oxidative and non-oxidative mechanisms (7, 8). Given the importance of macrophages and neutrophils in the immune response, it is reasonable to further investigate the immune mechanisms of these cells exposed to *C. neoformans*.

Long non-coding RNAs (lncRNAs) are non-coding RNAs with a length greater than 200 nucleotides that lack protein-coding potential and participate in the regulation of immune cell differentiation by binding with DNA, RNA, or proteins (9, 10). It was reported that lncRNAs derived from *C. neoformans* regulate fungal morphological changes (11), but the regulation of *C. neoformans* infection by lncRNAs from host cells has not been widely reported. LincR-PPP2R5C has recently been found to regulate type 2 immune responses in asthma (12). *C. neoformans* induces type 2 immune responses during infection in mice, which is detrimental to host protection (13–17). However, whether LincR-PPP2R5C participates in the regulation of pulmonary *C. neoformans* infection is unclear. In the present study, we showed that LincR-PPP2R5C deficiency mitigated pulmonary *C. neoformans* infection in mice, and we further investigated the mechanisms of type 2 cytokine release and its protective effects against pulmonary *C. neoformans* infection in mice deficient in LincR-PPP2R5C.

## MATERIALS AND METHODS

### Mice

Wild-type (WT) C57BL/6J mice were purchased from the laboratory animal center of Nanjing Medical University (Nanjing, China). LincR-PPP2R5C knockout (KO) mice were generated by Cyagen Biosciences (#KOCMS170904JN1; California, USA) using CRISPR/Cas9 techniques. Briefly, gRNA to the mouse LincR-PPP2R5C gene and Cas9 mRNA were microinjected into fertilized mouse eggs to obtain F0 generation mice. F0 founders were confirmed by PCR genotyping and then crossed with WT mice to generate F1 offspring. The KO mice used in this study were generated by backcrossing the F1 generation with C57BL/6J mice for more than ten generations. The animals were housed under specific pathogen-free conditions at Nanjing Medical University. All mice used in the study were female and between 6 and 8 weeks old, and all the animal studies were conducted with sex- and age-matched mice.

### *C. neoformans* strains and culture

*C. neoformans* strain H99 (208821, ATCC) was first removed from −80°C and activated on Sabouraud dextrose agar (SDA, 210950; Becton Dickinson) plates. Individual colonies from agar plates were cultured overnight in 10 mL of Sabouraud dextrose broth (238230, Becton Dickinson) at 30°C and 200 rpm. The next day, yeast cells were counted using a hemocytometer and diluted to the desired concentration in sterile phosphate-buffered saline (PBS).

### Intratracheal infection

Seven-week-old female WT or LincR-PPP2R5C KO mice were anesthetized with pentobarbital sodium by intraperitoneal injection and then intratracheally challenged with *C. neoformans* strain H99 (1 × 10^4^ CFU) in 30 µL of PBS. For survival analysis, the mice were monitored daily after infection with *C. neoformans*. For the cryptococcal organ burden assay, the mice were sacrificed at 21 days post infection. Some mice were sacrificed at 14 days or 48 h post infection.

In some experiments, mice received daily intraperitoneal injections of anti-interleukin (IL)-4 (BP0045, BioXcell), anti-CD4 (BE0003, BioXcell), anti-Ly6G (BE0075, BioXcell), or isotype control antibody starting on day 7 or 14 post infection. The mice were then sacrificed on day 14 or 21 post infection, and the organs of the mice were removed for fungal burden detection.

### Bronchoalveolar lavage fluid harvest

After the mice were anesthetized, the trachea of the mice was fully exposed, and a 22-gauge catheter was inserted into the trachea of the mice for fixation. Five hundred microliters of cold PBS containing 1-mM EDTA was injected through the catheter. After 5 seconds, the solution was aspirated gently, and the extracted fluid was collected. The above steps were repeated twice with a recovery rate of more than 80%, and approximately 1.2 mL of mouse alveolar lavage fluid was finally obtained. The bronchoalveolar lavage fluid (BALF) was then centrifuged at 500 × *g* for 5 min, and the supernatants were stored at −80°C for subsequent enzyme-linked immunosorbent assay (ELISA) detection, while the cell precipitate was suspended in PBS, and then the cell counts were determined using a hemocytometer.

### Detection of fungal burden

The mice were sacrificed at 21 and 14 days or 48 h post infection, and the lung, spleen, and brain were removed for cryptococcal organ burden detection as previously described (18). Briefly, the lung tissue was homogenized sufficiently in 1 mL of sterile PBS and then diluted 100 times with PBS. Ten-microliter dilutions of homogenates were inoculated onto SDA plates, and six replicates were performed for each sample. Subsequently, the SDA plates were cultured at 32°C for 36 h, after which the number of colonies was counted.

### Histopathological analysis

After euthanasia, the mice were dissected to expose their hearts and lungs. Cold PBS was injected into pulmonary blood vessels from the right ventricle to clear blood from the lungs. The inferior lobes of the right lungs were removed, fixed in 4% formalin, paraffin-embedded, and cut into 5-µm-thick tissue sections. After that, the sections were stained with hematoxylin-eosin (H&E), periodic acid-Schiff (PAS), and mucicarmine reagents and photographed by optical microscopy.

### Quantitative real-time PCR

Total RNA was extracted from 10 mg of lung tissue using TRIzol reagent (Thermo Fisher Scientific) according to the manufacturer’s instructions. The concentration of the extracted RNA was further measured by a NanoDrop One Microvolume UV-Vis spectrophotometer (Thermo Fisher Scientific). Subsequently, RNA was reverse-transcribed into cDNA using reverse-transcription reagents (11141ES60, Yeasen). Quantitative real-time PCR (qRT‒PCR) was conducted on a StepOnePlus Real-Time PCR System (ABI, USA) using SYBR Green qPCR master mix (11184ES08, Yeasen). Individual mRNA abundance was quantified by cycle threshold (CT) values and normalized to the β-actin mRNA level. The relative expression of the target genes refers to the fold change in the relative expression level of the experimental group compared with that of the control group. The sequences of the primers used in this study are listed in Table 1.

**TABLE 1:** Primers in the study

Name	Sequence
β-Actin-F	GAGAAGCTGTGCTATGTTGCT
β-Actin-R	CTCCAGGGAGGAAGAGGATG
LincR-PPP2R5C-F	AGTCTCGTAAGGTTGATTGGATG
LincR-PPP2R5C-R	CACATTTTACTACGAGCAACGGA

### Enzyme-linked immunosorbent assay

Commercially available IL-4 (431104, BioLegend), IL-5 (431204, BioLegend), tumor necrosis factor-alpha (TNF-α) (430916, BioLegend), and IL-6 (431315, BioLegend) ELISA kits were used to detect cytokines in lung tissue homogenates, and a Mouse NE/ELA2 (Neutrophil Elastase/Elastase-2) ELISA kit (E-EL-M3025, Elabscience) was used to quantify elastase in the cell lysates, BALF, and lung tissue homogenates. All operations were performed according to the manufacturer’s instructions.

### Flow cytometry

Single-cell suspensions of lung tissues were first stained with a Zombie NIR Fixable Viability Kit (423106, Biolegend), and then Fc receptors were blocked with an anti-CD16/32 antibody (Clone: 93, 101302; BioLegend). The cells were further incubated with the following antibodies: eFluor 450-conjugated anti-mouse CD3 (Clone: 17A2, 48–0032-82; Invitrogen), fluorescein isothiocyanate (FITC)-conjugated anti-mouse CD4 (Clone: GK1.5, 11–0041-82; eBioscience), phycoerythrin (PE)-conjugated anti-mouse IL-4 (Clone: 11B11, 504104; BioLegend), PE-conjugated rat IgG1 isotype control (Clone: R3-34, 554685; BD Biosciences), BV421-conjugated anti-mouse F4/80 (Clone: BM8, 123137; BioLegend), APC-conjugated anti-mouse Arg1 (Clone: A1exF5, 17–3697-82; Invitrogen), PE-Cy7-conjugated anti-mouse MHCII (Clone: M5/114.15.2, 107630; BioLegend), PE-conjugated anti-mouse CD11c (Clone: N418, 12–0114-81; Invitrogen), FITC-conjugated anti-mouse CD11b (Clone: M1/70, 11–0112-81; Invitrogen), and APC-conjugated anti-mouse Ly6G. Notably, a membrane-breaking reagent (554715, BD Biosciences) was used before PE-conjugated anti-mouse IL-4 was used for labeling. In addition, counting beads (01–1234-42, eBioscience) were added to count the cells. Ultimately, the stained cells were detected and recorded by a BD FACSCalibur Flow Cytometer (BD Bioscience), and the data were analyzed using FlowJo software (Treestar, Woodburn, USA).

### Western blot analysis

After being stimulated with *C. neoformans* (5 × 10^6^ CFU) alone or *C. neoformans* (5 × 10^6^ CFU) combined with recombinant mouse IL-4 protein (15 ng/mL) for 4 h, neutrophils (1 × 10^6^) were harvested and washed twice with cold PBS. The cells were then lysed in radioimmunoprecipitation assay (RIPA) buffer (P0013B, Beyotime Biotech) containing phenylmethanesulfonyl fluoride (PMSF) (ST506, Beyotime Biotech) for 10 min. The cells were further disrupted with an ultrasonic cell disruptor and centrifuged at 12,000 revolutions per minute (RPM) and 4°C for 10 min. The supernatant was transferred to 1.5-mL Eppendorf tubes and denatured in 5× SDS-PAGE loading buffer (20315ES, Yeasen) at 100°C for 10 min. The protein samples were separated by 10% polyacrylamide gel electrophoresis and transferred to a polyvinylidene diﬂuoride membrane (Merck Millipore) with a pore size of 0.45 µm. The blots were then blocked with 5% non-fat dried milk in Tris-buffered saline supplemented with 0.05% Tween 20 (TBST) and incubated with primary antibodies against β-actin (1:2,000, GB15003; Servicebio), total protein phosphatase 2A (PP2A) (1:5,000, ab32104; Abcam), phosphorylated PP2A (1:1,000, sc-271903; Santa Cruz Biotechnology), methylated PP2A (1:1,000, sc-81603; Santa Cruz Biotechnology), and neutrophil elastase (NE) (1:1,000, ab68672; Abcam) overnight. The membranes were washed in TBST and incubated with horseradish peroxidase-conjugated anti-rabbit IgG secondary antibody (1:10,000, 7074; Cell Signaling Technology) or anti-mouse IgG secondary antibody (1:5,000, 7076; Cell Signaling Technology) at room temperature for 1 h. Proteins were detected by chemiluminescence using enhanced chemiluminescence (36208-A, Yeasen) in a Tanon 5200 Multi Automatic Chemiluminescence/Fluorescence Image Analysis System and were quantified by ImageJ software.

### Neutrophil isolation

Mouse neutrophils were isolated from bone marrow as previously reported (19). After euthanasia, the femurs and tibias of the mice were collected in sterile PBS. The ends of the femurs and tibias were cut with scissors, and then the bone marrow was flushed with a 1-mL sterile syringe filled with PBS and filtered through a 70-µm cell strainer (Falcon) into a 50-mL centrifuge tube. Then, the cell suspension was washed by centrifugation and resuspended in 1 mL of sterile PBS. Three milliliters of Histopaque 1119 (11191, Sigma–Aldrich), 3 mL of Histopaque 1077 (10771, Sigma–Aldrich), and 1 mL of cell suspension were slowly and gently added to a 15-mL centrifuge tube in sequence and then centrifuged at 800 × *g* for 30 min at room temperature without breaking. Finally, neutrophils were collected from the intermediate layer of 1119 and 1077 using a clean transfer pipette and were transferred to a 15-mL centrifuge tube. After washing twice with PBS, the obtained cells were resuspended in RPMI 1640 medium supplemented with 10% FBS, counted, and seeded into cell culture plates at the appropriate concentrations.

### Bone marrow-derived macrophage culture

The isolation and culture of bone marrow-derived macrophages were performed as follows: after euthanasia, the femurs and tibias of the mice were collected in sterile PBS. The ends of the femurs and tibias were cut with scissors, and then the bone marrow was flushed with a 1-mL sterile syringe filled with PBS and filtered through a 70-µm cell strainer (Falcon) into a 50-mL centrifuge tube. Subsequently, the cell suspension was washed by centrifugation and resuspended in 1 mL of sterile red blood cell lysis buffer (00–4333-57, Invitrogen). After 5 min, the cells were washed twice with sterile PBS. Then, the bone marrow cells were seeded at a density of 1 × 10^6^ cells/mL in a 24-well plate with Dulbecco’s modified Eagle’s medium (DMEM) containing 20-ng/mL granulocyte-macrophage colony-stimulating factor (GM-CSF). On day 3, 0.5 mL of culture medium was discarded, and the medium was replaced with an additional 0.5 mL of fresh medium. On day 6, the culture medium was completely replaced. On day 7, the cells were harvested for subsequent experiments.

### *In vitro* killing test

Neutrophils or macrophages from 7-week-old female WT or LincR-PPP2R5C KO mice were cultured with *C. neoformans* in 96-well cell culture plates at a multiplicity of infection (MOI) = 0.01. Recombinant mouse IL-4 protein (abs04101, Absin) was added to the experimental group at a concentration of 15 ng/mL. In some experiments, alvelestat (A12779, ADOOQ) was used to inhibit neutrophil elastase at the recommended concentrations, and dimethyl sulfoxide (DMSO) was used as a control. The total culture volume was 200 µL. After coculturing for 4 h, the neutrophils were transferred to 1.5-mL Eppendorf tubes and lysed by exposing them to distilled water so that the pellets inside the cells could be completely released. The diluted culture medium was then inoculated on the SDA plate and incubated at 32°C for 36 h to obtain the number of CFUs.

### ROS measurement

The level of intracellular reactive oxygen species (ROS) was detected by using a reactive oxygen species assay kit (S0033S, Beyotime) (20). In brief, cells were seeded at a density of approximately 10,000 cells/well in 96-well cell culture plates. After stimulation, the ROS detection reagent was added to the wells and incubated at 37°C for 1 h. The ROS level was detected by a BD FACSCalibur Flow Cytometer (BD Biosciences), and the mean fluorescence intensity (MFI) was analyzed using FlowJo software (Treestar).

### Statistical analysis

Statistical analyses were conducted using GraphPad Prism version 9. All the data are presented as means ± SEMs. One-way analysis of variance was used to compare the means of three or more independent groups. An unpaired *t*-test was used to evaluate the differences between two groups. Survival curves were analyzed using the log-rank test. Statistical significance was determined as follows: *P < 0.05, **P < 0.01, ***P < 0.001, ****P < 0.0001, and ns, which means “not signiﬁcant.”

## RESULTS

### LincR-PPP2R5C deficiency mitigated pulmonary cryptococcosis

**Fig 1:**
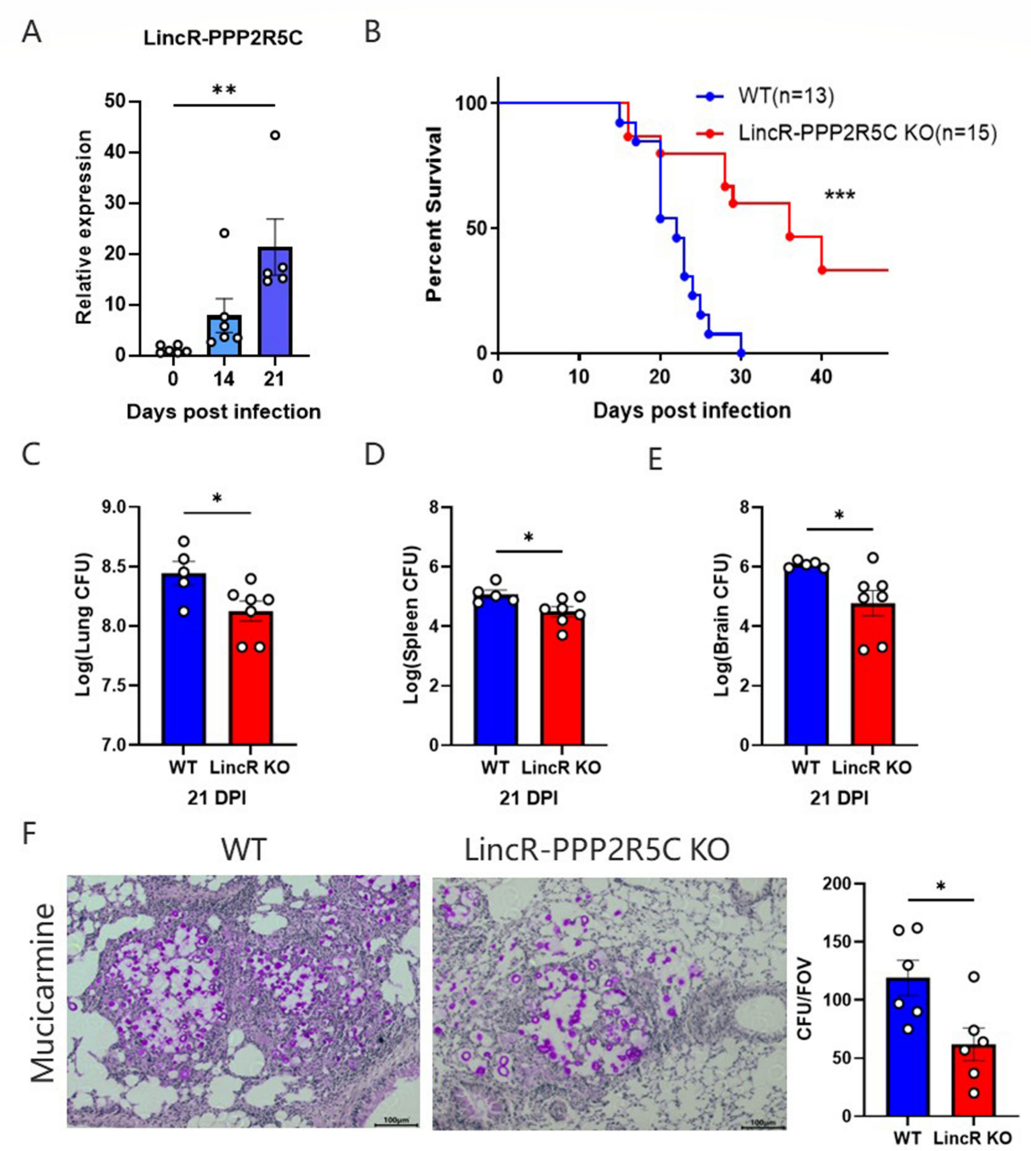
LincR-PPP2R5C deficiency mitigated pulmonary *C. neoformans* infection. (**A**) Lung RNA was extracted at 0, 14, and 21 days post infection (DPI) and reverse transcribed, and LincR-PPP2R5C gene expression was normalized to that of the β-actin gene using the 2^−ΔΔCt^ method. The data are shown as the mean ± SEM (*n* = 5–6 mice per group). ** *P* <0.01, using ANOVA. (**B**) WT (*n* = 13) and LincR-PPP2R5C KO (*n* = 15) mice were intratracheally inoculated with 1 × 10^4^ *C. neoformans* strain H99 and monitored daily for survival. ***P < 0.001, using a log-rank test. (**C–E**) Lung, spleen, and brain CFU were counted at 21 days post infection by plating lung homogenates on SDA. The data are shown as the mean ± SEM (*n* = 6 mice per group). *P < 0.05, unpaired Student’s *t*-test. (**F**) Representative lung sections were stained with mucicarmine, and *C. neoformans* spp. are indicated as red-stained cells surrounded by a clear capsular halo. The figure on the right represents a quantitative analysis of *C. neoformans* in each field of view.

To determine whether LincR-PPP2R5C was altered in pulmonary *C. neoformans* infection, we examined LincR-PPP2R5C in lung tissue at different times after the mice were infected with *C. neoformans*. As shown in Fig. 1A, the level of LincR-PPP2R5C in the lung tissues increased significantly at 14 days post infection and peaked at 21 days post infection, indicating that LincR-PPP2R5C may be involved in pulmonary *C. neoformans* infection. To further investigate the role of LincR-PPP2R5C in pulmonary *C. neoformans* infection, we tested whether LincR-PPP2R5C deficiency affected the survival of infected mice, and the results showed that LincR-PPP2R5C deficiency had a protective effect on mice following exposure to *C. neoformans* (Fig. 1B). The median survival time of the LincR-PPP2R5C KO mice was notably longer than that of the WT mice. Furthermore, we quantified the organ dissemination of fungi in the lung, spleen, and brain tissues of WT mice and LincR-PPP2R5C KO mice following intratracheal inoculation with *C. neoformans*. Compared with that in WT mice, the fungal burden in LincR-PPP2R5C KO mice was significantly lower (Fig. 1C through E). Mucicarmine staining was performed to observe fungi in the lung tissues. As shown in Fig. 1F, the pulmonary fungal burden in the LincR-PPP2R5C KO-infected mice was significantly reduced. Overall, LincR-PPP2R5C deficiency had a protective effect on mice with pulmonary *C. neoformans* infection, suggesting that LincR-PPP2R5C may be involved in the regulation of pulmonary *C. neoformans* infection.

### Pathological observations of pulmonary tissues infected with *C. neoformans*

**Fig 2:**
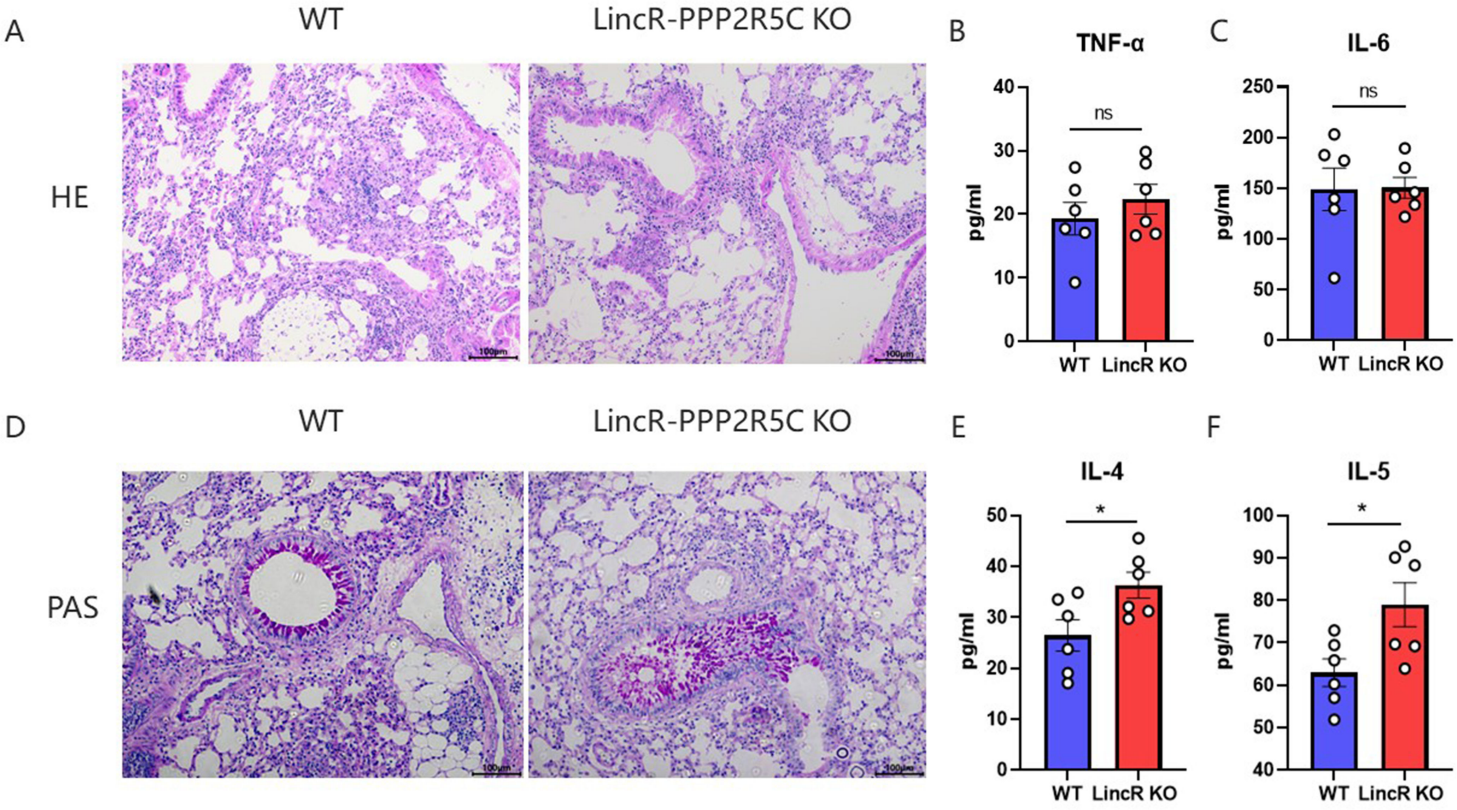
Pathological observations of lung tissues from patients with pulmonary cryptococcosis. WT and LincR-PPP2R5C KO mice were infected intratracheally with 1 × 10^4^ *C. neoformans* strain H99 and sacrificed at 21 days post infection. (**A**) Representative lung sections were stained with hematoxylin-eosin (HE) to analyze the infiltration of inflammatory cells. TNF-α (**B**) and IL-6 (**C**) in the lung homogenates were detected by ELISA. (**D**) Representative lung sections were stained with PAS to show goblet cell hyperplasia. IL-4 (**E**) and IL-5 (**F**) in the lung homogenates were detected by ELISA. The data are expressed as the mean ± SEM (*n* = 6 mice per group). *P < 0.05, unpaired Student’s *t*-test. ns, not significant.

Considering that the differences in survival curves and lung CFUs between WT mice and LincR-PPP2R5C KO mice may be related to lung tissue pathology, we performed H&E and PAS staining on lung sections from WT mice and LincR-PPP2R5C KO mice at 21 days post infection. As shown in Fig. 2A, H&E staining of the lung sections demonstrated no differences between the groups. We then examined the levels of TNF-α and IL-6, which are known to be associated with inflammation, and consistently, TNF-α and IL-6 were similar in WT mice and LincR-PPP2R5C KO mice after *C. neoformans* infection (Fig. 2B and C). However, PAS staining revealed more airway goblet cell metaplasia in LincR-PPP2R5C KO mice than in WT mice (Fig. 2D). Since IL-4 and IL-5 have previously been reported to promote mucus secretion in asthmatic patients (21), we examined their expression levels in the lung tissues of WT and LincR-PPP2R5C KO mice at 21 days post infection. IL-4 and IL-5 levels were significantly greater in LincR-PPP2R5C KO mice than in WT mice (Fig. 2E and F), suggesting that LincR-PPP2R5C regulates pulmonary *C. neoformans* infection, which may be at least partially mediated by IL-4 and IL-5.

### LincR-PPP2R5C deficiency increased type 2 cytokine levels in pulmonary cryptococcosis

**Fig 3:**
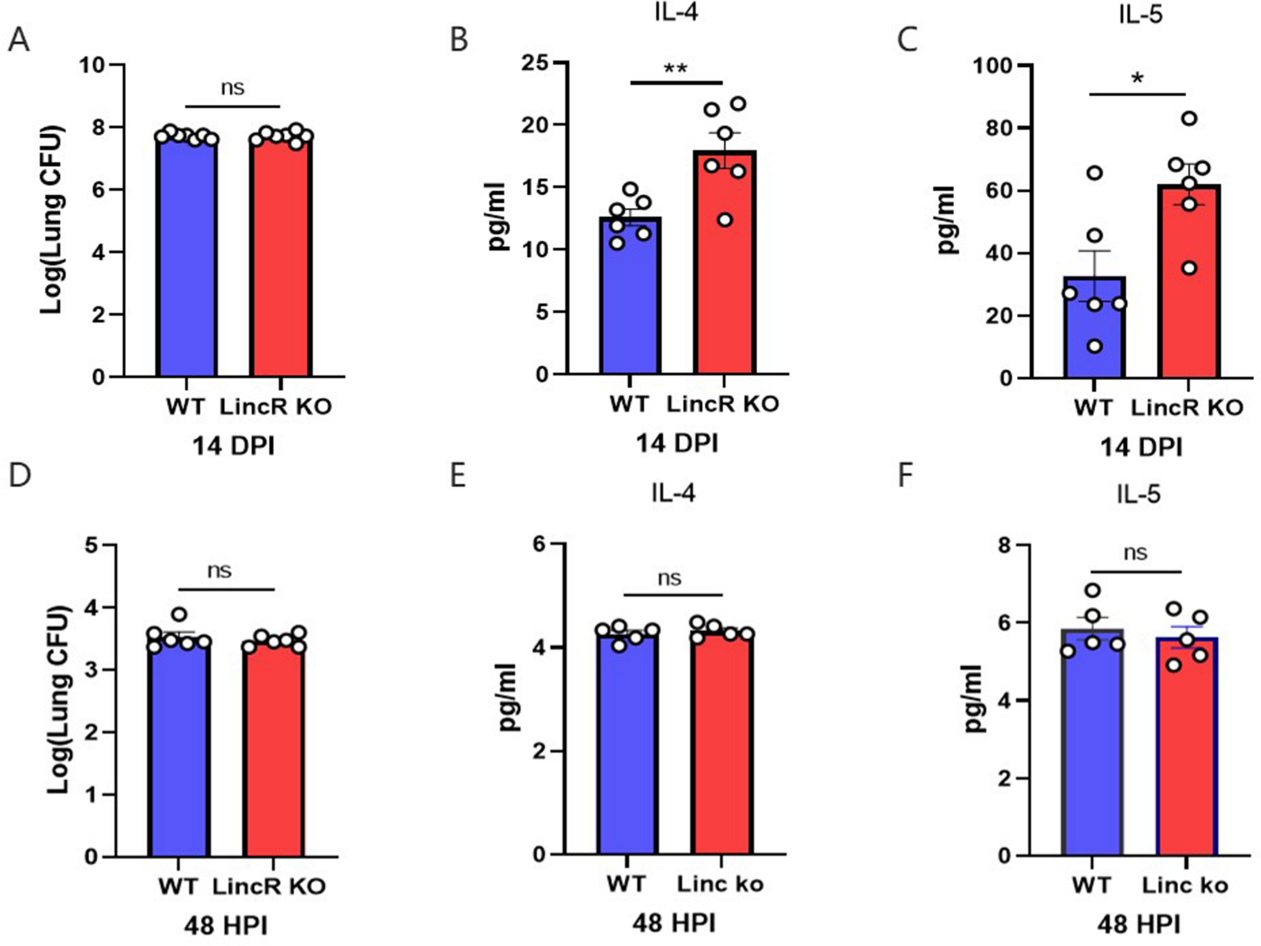
LincR-PPP2R5C deficiency increased IL-4 and IL-5 expressions. WT and LincR-PPP2R5C KO mice were infected intratracheally with 1 × 10^4^ *C. neoformans* strain H99. (**A**) Mice were sacrificed at 14 days post infection. Lung CFUs were assessed by plating lung homogenates on SDA, and the levels of IL-4 (**B**) and IL-5 (**C**) in the lung homogenates were detected via ELISA. (**D**) Mice were sacrificed at 48 h post infection. Lung CFUs were assessed by plating lung homogenates on SDA, and the levels of IL-4 (**E**) and IL-5 (**F**) in the lung homogenates were detected via ELISA. The data are presented as means ± SEMs (*n* = 5–7 per group). *P < 0.05, **P < 0.01; unpaired Student’s *t* test. ns, not significant.

Both innate and adaptive immune cells can produce IL-4 and IL-5 (22). Usually, innate immune responses develop at the early stage, and adaptive immune responses follow at the later stage. To investigate whether increases in IL-4 and IL-5 occurred in LincR-PPP2R5C KO mice infected with *C. neoformans*, the fungal burden in lung tissue and the levels of IL-4 and IL-5 were detected in WT and LincR-PPP2R5C KO mice at 14 days post infection and 48 h post infection, respectively. As shown in Fig. 3A, although the fungal burden in the lung tissue was not significantly different among the groups, the levels of IL-4 and IL-5 were strikingly increased in the LincR-PPP2R5C KO mice at 14 days post infection (Fig. 3B and C). In contrast, the CFU and IL-4 and IL-5 concentrations in the lung tissues of the WT and LincR-PPP2R5C KO mice remained low at 48 h post infection, and no difference was detected between the two groups (Fig. 3D through F). In conclusion, the increase in IL-4 and IL-5 induced in LincR-PPP2R5C KO-infected mice may be dependent on the adaptive immune response.

### LincR-PPP2R5C deficiency enhanced the secretion of IL-4 by non-T cells

CD4^+^ T cells in the adaptive immune response are the main source of IL-4 (23). Therefore, to explore the source of IL-4 in LincR-PPP2R5C KO-infected mice, flow cytometry was used to detect the expression level of IL-4 in T cells and non-T cells in the lung tissues of WT and LincR-PPP2R5C KO mice at 21 days post infection. The gating strategy for IL-4^+^ cells is depicted in Fig. S1A, in which most IL-4^+^ cells were CD3^−^CD4^−^, suggesting that non-T cells may dominate the production of IL-4. Statistical analysis revealed that LincR-PPP2R5C deficiency increased the percentage and number of IL-4^+^ cells, although the difference was not significant (Fig. S1B and C). To further verify the source of IL-4, we analyzed CD3^+^ cells and found that IL-4 expression in CD3^+^ cells and CD3^+^CD4^+^ cells was quite rare in both WT mice and LincR-PPP2R5C KO mice infected with *C. neoformans* (Fig. S1D through F). Collectively, these data suggested that the increased IL-4 in the infected LincR-PPP2R5C KO mice was predominantly derived from non-T cells.

We further validated the above experimental results through an *in vivo* experiment involving CD4^+^ cell depletion. A schematic of the infection and drug administration methods is shown in Fig. S2A. The results showed that the depletion of CD4^+^ cells had no effect on the expression level of IL-4 in *C. neoformans*-infected mouse lungs (Fig. S2B and C), suggesting that IL-4 might originate from non-T cells. In addition, upon the administration of antibodies, the levels of pulmonary IL-4 in WT and LincR-PPP2R5C KO mice seemed comparable, suggesting that IL-4-producing cells may be influenced by non-specific interference via FcγR, which is bound with IgG2b or the anti-CD4.

### Blocking IL-4 in the absence of LincR-PPP2R5C did not reduce the pulmonary fungal burden in mice

**Fig 4:**
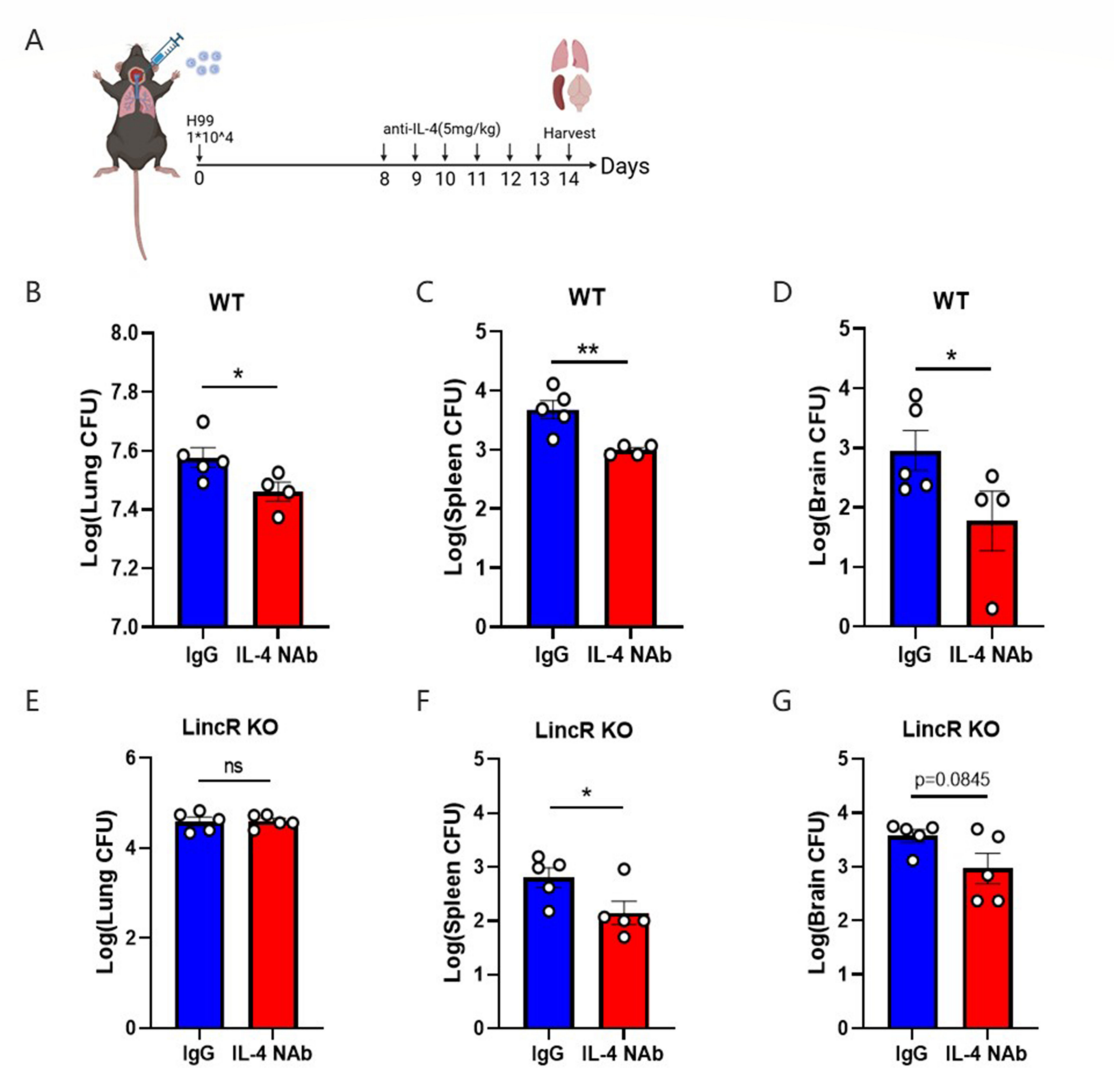
Blocking IL-4 reduced the pulmonary fungal burden in WT mice but not in LincR-PPP2R5C KO mice. WT and LincR-PPP2R5C KO mice were challenged intratracheally with 1 × 10^4^ CFU of *C. neoformans* strain H99 and were given InVivoMAb against IL-4 and a rat IgG1 isotype control, respectively, at 7 days post infection (100 µg/day for 7 consecutive days). The mice were then sacrificed at 14 days post infection. (**A**) Schematic of drug administration for mice infected with *C. neoformans*. (**B–D**) CFUs in the lungs, spleens, and brains of WT mice. (**E–G**) CFUs in the lungs, spleens, and brains of LincR-PPP2R5C KO mice. The data are presented as means ± SEMs (*n* = 4–5 mice per group). *P < 0.05, **P < 0.01; unpaired Student’s *t*-test.

Fewer CFUs were found in the lungs of IL-4 KO mice than in those of WT mice following exposure to *C. neoformans*, as shown in the study of Blackstock and Murphy (24), which implied that IL-4 may have an adverse effect on the body’s resistance to *C. neoformans* infection. To better understand the role of IL-4 in mice, we neutralized IL-4 in WT and LincR-PPP2R5C KO mice with an IL-4 neutralizing antibody and then measured the fungal burden in the organs of infected mice at 14 days post infection (Fig. 4A). The results confirmed that the fungal burdens in the lungs, spleens, and brains were significantly decreased in the WT mice with IL-4 blockade (Fig. 4B through D), which was consistent with the harmful effects of IL-4 reported in previous studies (15, 24). Surprisingly, however, there was no significant difference in fungal burden in the lungs of LincR-PPP2R5C KO mice with IL-4 neutralization, although the number of CFUs appeared to decrease in the spleen and brain (Fig. 4E through G). These results suggested that in LincR-PPP2R5C KO-infected mice, IL-4 may have dual roles: being detrimental in the spleen and brain and dispensable in the lung.

### LincR-PPP2R5C deficiency altered lung macrophages in infected mice

**Fig 5:**
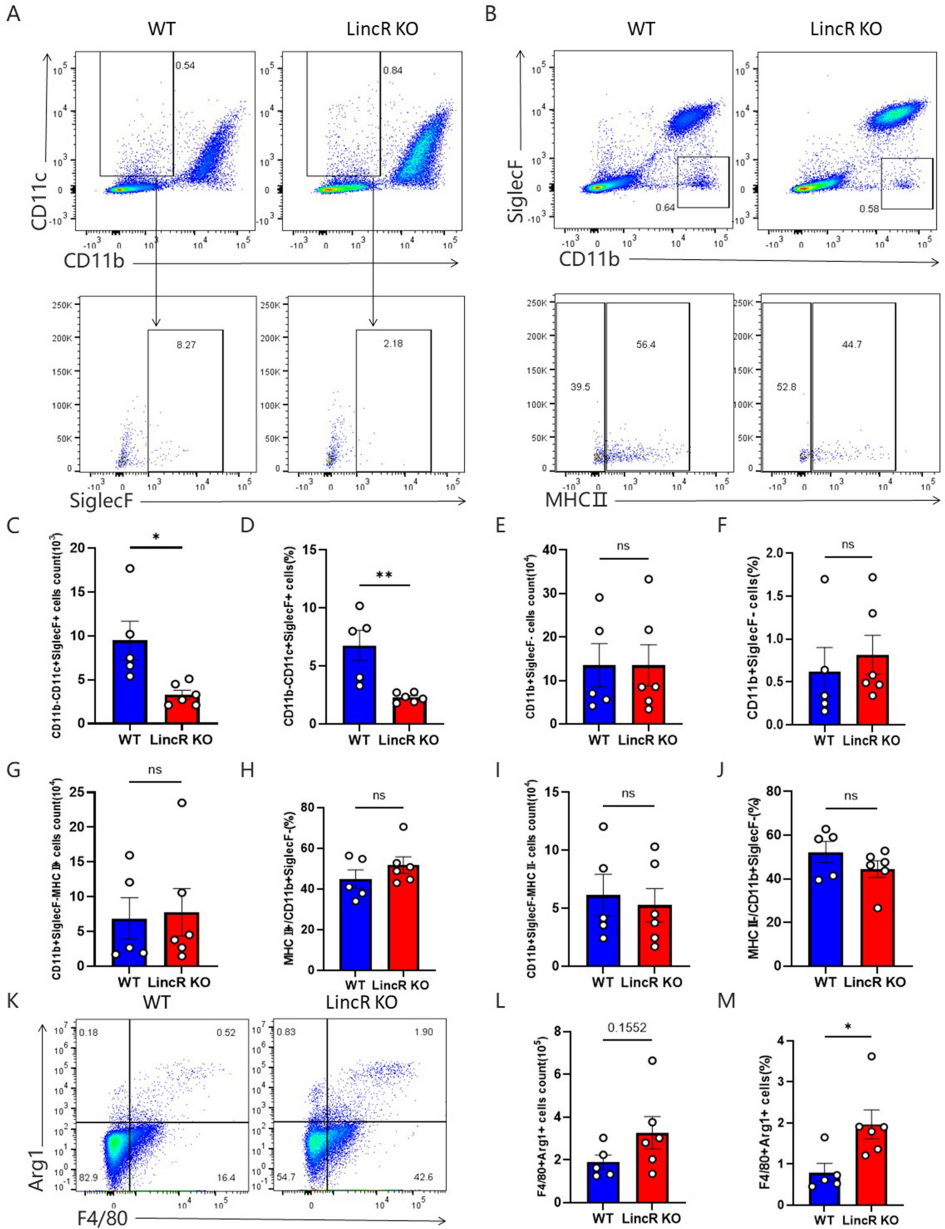
Comparison of macrophage infiltration and polarization in infected WT and LincR-PPP2R5C KO mice. WT and LincR-PPP2R5C KO mice were infected intratracheally with 1 × 10^4^ CFU of *C. neoformans* strain H99 and sacrificed at 21 days post infection. Total lung cells were analyzed by flow cytometry. (**A**) Representative plots of gating strategies for the identification of CD11b^−^CD11c^+^SiglecF^+^ alveolar macrophages. (**B**) Representative plots of gating strategies for the identification of CD11b^+^SiglecF^−^ interstitial macrophages. (**C**) Total number of CD11b^−^CD11c^+^SiglecF^+^ cells in the lung tissue. (**D**) Percentage of CD11b^−^CD11c^+^SiglecF^+^ cells among the total lung cells. (**E**) Total number of CD11b^+^ SiglecF^−^ cells in the lung tissue. (**F**) Percentage of CD11b^+^SiglecF^−^ cells among the total lung cells. (**G**) Total number of CD11b^+^SiglecF^−^MHCII^+^ cells in the lung tissue. (**H**) Percentage of CD11b^+^SiglecF^-^MHCII^+^ cells among CD11b^+^SiglecF^-^ cells. (**I**) Total number of CD11b^+^SiglecF^-^MHCII^-^ cells in the lung tissue. (**J**) Percentage of CD11b^+^SiglecF^−^MHCII^−^ cells among CD11b^+^SiglecF^−^ cells. (**K**) Representative gating strategy for F4/80^+^Arg1^+^ macrophages. (**L**) Total number of F4/80^+^Arg1^+^ cells in the lung tissue. (**M**) Percentage of F4/80^+^Arg1^+^ cells among the total lung cells. The data are presented as the mean ± SEM (*n* = 5–6 mice per group). *P < 0.05, **P < 0.01; unpaired Student’s *t* test. ns, not significant.

Valid control of *C. neoformans* growth in mouse lungs requires the activation of specialized phagocytes, such as macrophages, which kill *C. neoformans* through an inducible NO synthase-dependent mechanism (25). Macrophages are the most abundant immune cells in a healthy lung, and their presence is demarcated by distinct microenvironments: alveolar macrophages (AMs), which are located in the alveoli and airways, and interstitial macrophages (IMs), which are located within the lung parenchyma. Alveolar and interstitial lung macrophages exhibit different origins and lifespans in the lung and are closely related to infection and lung injury (26). To investigate the response of pulmonary macrophages to *C. neoformans*, flow cytometry was conducted on single-cell suspensions derived from the lung tissues of WT and LincR-PPP2R5C KO mice at 21 days post infection with *C. neoformans*. The gating strategy used for flow cytometry analysis of lung macrophages is depicted in Fig. 5A and B. Notably, in LincR-PPP2R5C KO mice, both the absolute number and percentage of AMs (CD11b^−^CD11c^+^SiglecF^+^) significantly decreased (Fig. 5C and D). However, no differences in the absolute number or proportion of IMs (CD11b^+^SiglecF^−^) were observed between WT and LincR-PPP2R5C KO mice (Fig. 5E and F). Further analysis of this population revealed that there were no significant differences in the absolute number or percentage of MHC-II^+^ or MHC-I^−^ macrophages between infected WT and LincR-PPP2R5C KO mice (Fig. 5G through J).

To further characterize the phenotypes of macrophages in the lungs of WT and LincR-PPP2R5C KO mice, a comparative analysis of F4/80^+^Arg1^+^ macrophages (M2) was performed using flow cytometry. The gating strategy is shown in Fig. 5K. At 21 days post infection, the number of M2 macrophages was increased in the LincR-PPP2R5C KO mice. The difference in the percentage of M2 macrophages between WT- and LincR-PPP2R5C KO-infected mice was significant (Fig. 5L and M). Taken together, these data revealed that, upon *C. neoformans* pulmonary infection, LincR-PPP2R5C deficiency decreased AMs, had no effect on the infiltration of lung IMs, and may induce M2 polarization.

### IL-4 activated the killing of *C. neoformans* by LincR-PPP2R5C KO neutrophils

**Fig 6:**
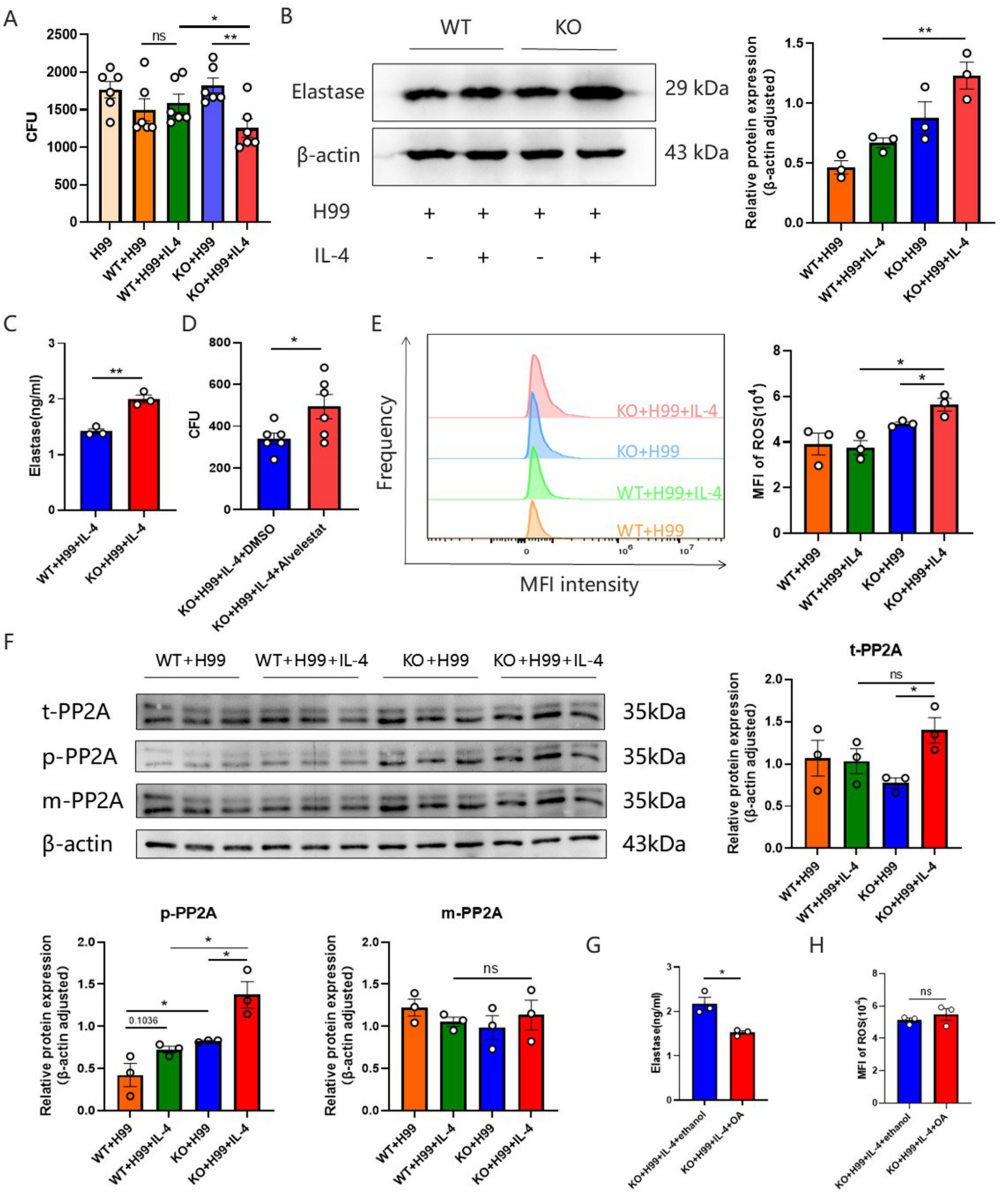
IL-4 activated LincR-PPP2R5C KO neutrophils to kill *C. neoformans*. (**A**) Fungicidal activity of WT and LincR-PPP2R5C KO neutrophils against *C. neoformans* stimulated with or without IL-4 (15 ng/mL) (*n* = 6 per group). (**B**) Western blot (WB) for elastase in WT and LincR-PPP2R5C KO neutrophils stimulated as described in panel **A** for 4 h. The data are representative of three independent experiments, and the statistical analysis is shown on the right. (**C**) ELISA detection of elastase levels in WT and LincR-PPP2R5C KO neutrophils cocultured with *C. neoformans* for 4 h under IL-4 stimulation. (**D**) Fungicidal activity of LincR-PPP2R5C KO neutrophils against *C. neoformans* after treatment with alvelestat or dimethyl sulfoxide. (**E**) MFI of ROS in WT and LincR-PPP2R5C KO neutrophils stimulated as described in panel** A** for 4 h, as detected by flow cytometry. The statistical diagram is shown on the right. (**F**) WB was used to detect the expression levels of total PP2A, phosphorylated PP2A, and methylated PP2A in WT and LincR-PPP2R5C KO neutrophils after coculture with *C. neoformans* for 4 h, with or without IL-4 stimulation. The statistical graph is shown on the right. Under the stimulation conditions depicted in panel **A**, the elastase content (**G**) and the MFI of ROS (**H**) in LincR-PPP2R5C KO neutrophils were detected by ELISA and flow cytometry, respectively, after treatment with okadaic acid (OA, 2 nM) or ethanol. The data are shown as mean ± SEM. *P < 0.05, **P < 0.01; unpaired Student’s *t*-test. ns, not significant.

It has been reported that IL-4 is an important molecule during the maturation of pre-myeloid cells to neutrophilic lineages and that IL-4 serves as a functional activator of mature neutrophils (27). We therefore hypothesized that elevated IL-4 in LincR-PPP2R5C KO mice enhances the ability of neutrophils to kill fungi. To verify this hypothesis, neutrophils from WT and LincR-PPP2R5C KO mice were isolated and cultured for an *in vitro C. neoformans* killing test. Consistent with our hypothesis, LincR-PPP2R5C KO neutrophils showed a significant increase in the ability to kill *C. neoformans* under stimulation with a certain concentration of IL-4, while WT neutrophils showed no increase in the ability to kill *C. neoformans* under stimulation with the same concentration of IL-4 (Fig. 6A). Concomitantly, an *in vitro* macrophage fungicidal assay revealed that stimulation with IL-4 negatively impacted the antifungal efficacy of macrophages derived from WT mice, while the fungicidal potential of LincR-PPP2R5C KO macrophages remained unaffected (Fig. S3A). These findings implied that, upon the elevation of IL-4, neutrophils may be the predominant cells mediating fungicidal functions in LincR-PPP2R5C KO mice during *C. neoformans* infection.

To further understand how LincR-PPP2R5C KO neutrophils kill *C. neoformans*, the level of elastase in LincR-PPP2R5C KO neutrophils was detected since NE is regarded as a major proteinase in primary granules in neutrophils that engage in microbicidal activity (28–30). The elastase content in the LincR-PPP2R5C KO neutrophils was greater than that in the WT neutrophils after stimulation with the same concentration of IL-4 (Fig. 6B and C). Moreover, after the application of alvelestat, an inhibitor of elastase, the ability of LincR-PPP2R5C KO neutrophils to kill fungi significantly decreased (Fig. 6D). The production of ROS by the phagocyte NADPH oxidase NOX2 plays an important role in host defense against microbial pathogens (31). Thus, we speculated that intracellular ROS accumulation was critical for the ability of LincR-PPP2R5C KO neutrophils to kill *C. neoformans*. To test this possibility, we examined intracellular ROS levels in WT mouse neutrophils and LincR-PPP2R5C KO neutrophils treated with *C. neoformans* in the absence or presence of IL-4. The results suggested that the mean fluorescence intensity of ROS in neutrophils from LincR-PPP2R5C KO mice was significantly greater than that in neutrophils from WT mice stimulated with the same concentration of IL-4 (Fig. 6E), suggesting that, upon *C. neoformans* infection, LincR-PPP2R5C deficiency combined with IL-4 increased ROS in neutrophils.

### LincR-PP2R5C regulated neutrophil elastase via the PP2A signaling pathway

Previous studies have demonstrated that LincR-PPP2R5C deficiency alleviates Th2-driven airway inflammation, which is associated with PP2A activity (12). PP2A, a predominant serine threonine protein phosphatase, plays a critical role in the innate immune response against infection and is implicated in diverse essential cellular processes, including the regulation of signaling cascades, cell cycle progression, and carcinogenesis (32). The phosphorylation and methylation of PP2A are significant regulatory mechanisms that modulate its activity and function. To further explore the mechanisms underlying the enhanced expression levels of elastase and ROS in LincR-PPP2R5C KO neutrophils stimulated with IL-4 and infected with *C. neoformans*, Western blotting was conducted to assess the levels of total PP2A, phosphorylated PP2A, and methylated PP2A in the neutrophils. Compared to IL-4-treated WT neutrophils, IL-4-treated LincR-PPP2R5C KO neutrophils exhibited increased levels of PP2A phosphorylation, whereas no significant differences in the methylation levels or total PP2A content were detected (Fig. 6F). Statistical analysis also showed that IL-4 stimulation enhanced total PP2A and PP2A phosphorylation in infected LincR-PPP2R5C KO neutrophils (Fig. 6F). These findings suggested that the absence of LincR-PPP2R5C may augment the enzymatic activity of PP2A in neutrophils, in which IL-4 may play a synergistic role. To ascertain whether PP2A activity indeed plays a critical role in the production of elastase and ROS in LincR-PPP2R5C KO neutrophils, we supplemented the experimental conditions with a PP2A inhibitor (OA). The results revealed that the inclusion of OA markedly decreased elastase levels in LincR-PPP2R5C KO neutrophils (Fig. 6G). Conversely, the MFI of ROS remained unaffected by the addition of OA (Fig. 6H).

In summary, these findings indicated that LincR-PPP2R5C deficiency combined with IL-4 increased the phosphorylation of PP2A, thereby enhancing neutrophil elastase production and the fungicidal capacity of neutrophils against *C. neoformans*.

### LincR-PPP2R5C deficiency increased neutrophil infiltration in pulmonary cryptococcosis

**Fig 7:**
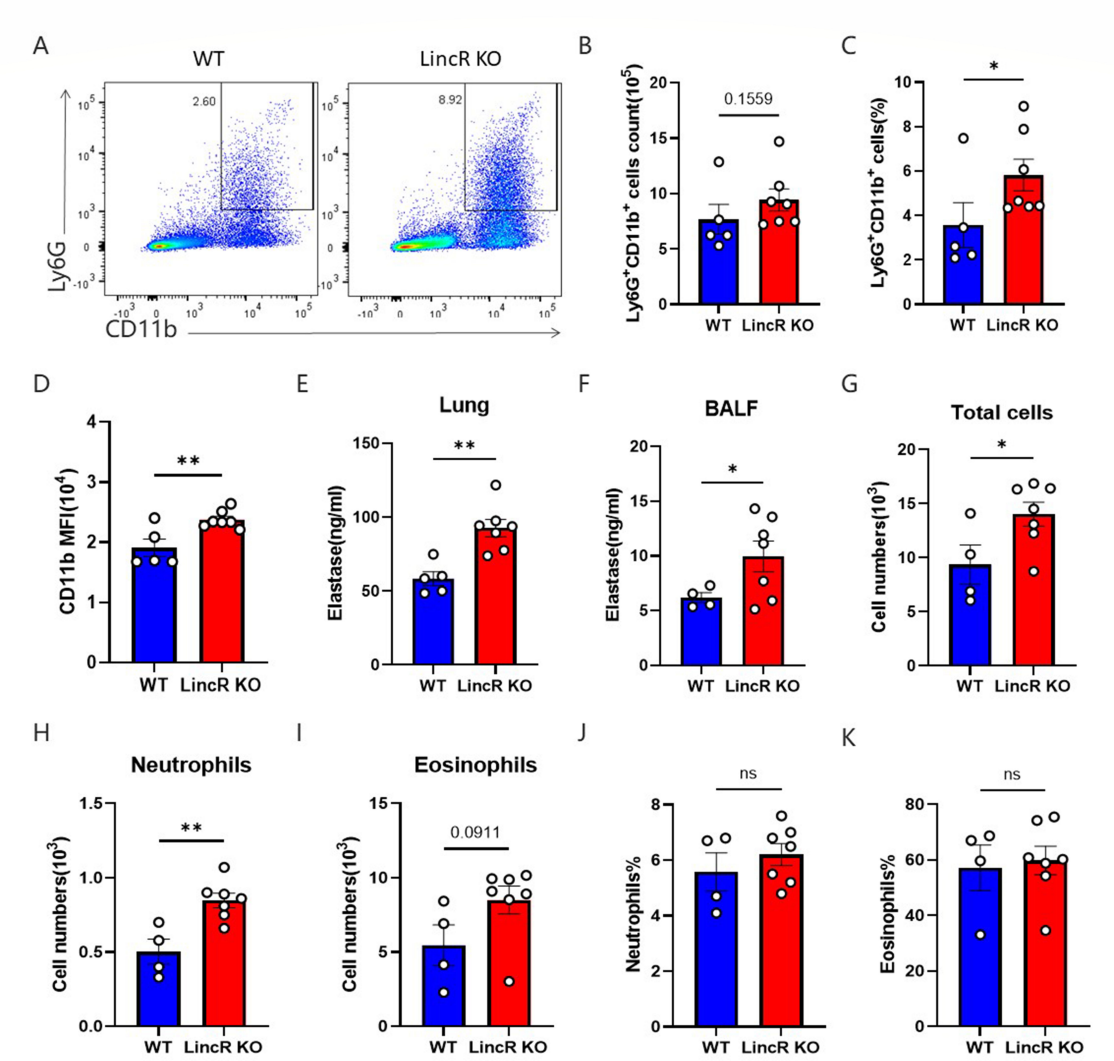
LincR-PPP2R5C deficiency rendered neutrophils more fungicidal against *C. neoformans* infection. WT and LincR-PPP2R5C KO mice were infected intratracheally with 1 × 10^4^ CFU of *C. neoformans* strain H99 and sacrificed at 14 days post infection. The BALF was harvested, and the lung cells were analyzed by flow cytometry. (**A**) Representative plots of the gating strategies used for Ly6G^+^CD11b^+^ cells. (**B**) Total numbers of Ly6G^+^CD11b^+^ cells per lung. (**C**) Percentage of Ly6G^+^CD11b^+^ cells among the total lung cells. (**D**) The MFI of CD11b is shown. (**E and F**) The concentration of elastase in lung tissue homogenates and BALF was determined using ELISA. (**G**) Total cell numbers in BALF. (**H**) The number of neutrophils in the BALF. (**I**) The number of eosinophils in the BALF. (**J**) The percentage of neutrophils among the total cells in the BALF. (**K**) The percentage of eosinophils among the total cells in the BALF. The data are shown as mean ± SEM (*n* = 6 mice per group). *P < 0.05, **P < 0.01; unpaired Student’s *t*-test. ns, not significant.

To determine whether the removal of *C. neoformans* was related to the maturation and enhanced killing ability of neutrophils in LincR-PPP2R5C KO mice, flow cytometry analysis of digested lung tissue (gate strategy, Fig. 7A) was performed, and immune cells in the BALF were analyzed. At 14 days post infection, compared to WT mice, LincR-PPP2R5C KO mice exhibited a significant increase in the percentage of Ly6G^+^CD11b^+^ neutrophils among the total cells in the lung tissue, although there was no significant difference in the absolute number of these cells between the two groups (Fig. 7B and C). Additionally, MFI analysis revealed that the CD11b MFI in the lung neutrophils of LincR-PPP2R5C KO mice was significantly greater than that in the lung neutrophils of WT mice (Fig. 7D). To verify whether neutrophil elastase plays a role in the killing of *C. neoformans in vivo*, the elastase content in lung homogenates and BALF was detected by ELISA. Compared to those of WT mice, the lung homogenates and BALF of LincR-PPP2R5C KO mice exhibited a significant increase in elastase content (Fig. 7E and F). Analysis of the BALF cells from both groups of mice revealed a significant increase in the total cell count and the number of neutrophils in the LincR-PPP2R5C KO mice (Fig. 7G and H), with a trend toward an increase in the number of eosinophils (Fig. 7I). However, no difference was observed in the proportion of neutrophils or eosinophils between the two groups (Fig. 7J and K). These results suggested that LincR-PPP2R5C deficiency increased neutrophil infiltration and increased elastase content in both lung tissue and BALF, thus limiting pulmonary *C. neoformans* growth. To further validate the role of neutrophils *in vivo*, we conducted a neutrophil depletion experiment in infected mice (Fig. S4A). Regrettably, injection of the anti-Ly6G antibody neither affected the fungal burden in the lung, spleen, or brain tissues of LincR-PPP2R5C KO mice (Fig. S4B through D) nor had an impact on the survival of the mice (Fig. S4E).

## DISCUSSION

Pulmonary cryptococcosis is a great challenge in immunocompromised individuals. Moreover, *C. neoformans*, the infectious fungus of pulmonary cryptococcosis, may cause pulmonary nodules in the healthy population. Innate immune cells, especially macrophages and neutrophils, constitute the first type of immune defense against pathogens. IL-4, a typical type 2 cytokine, usually dampens immune defense and amplifies infection. In the present study, we found that exposure to *C. neoformans* caused a marked increase in LincR-PPP2R5C in lung tissues. LincR-PPP2R5C deficiency significantly reduced pulmonary *C. neoformans* infection and improved the survival of mice. An investigation of the causes revealed that LincR-PPP2R5C deficiency led to an increase in the secretion of type 2 cytokines such as IL-4. It was also found that the deficiency of LincR-PPP2R5C collaborates with IL-4 to increase the phosphorylation level of PP2A, thus enhancing the fungicidal activity of neutrophils. Furthermore, following infection with *C. neoformans*, the infiltration of neutrophils into the lung tissue and BALF of LincR-PPP2R5C KO mice increased.

*C. neoformans* is a human invasive fungal pathogen that can be inhaled into the lungs through the respiratory tract. In immunocompromised individuals, *C. neoformans* can cause pneumonia, acute respiratory distress syndrome, and subsequent extrapulmonary transmission (3). During infection in mice, *C. neoformans* induces a type 2 immune response that is detrimental to host protection (13–17), which is very similar to asthma. Recently, knockout and conditional knockout of LincR-PPP2R5C significantly reduced Th2 differentiation and alleviated airway inflammation in a mouse model of acute asthma (12). However, whether LincR-PPP2R5C is involved in the regulation of pulmonary *C. neoformans* infection has not been clarified.

Previous studies have reported that IL-4 may originate from eosinophils, CD4^+^ T cells, and other cell types (23, 33). Our study demonstrated that IL-4 in the lung tissue of mice infected with *C. neoformans* originated from non-T cells but did not identify the specific cell type responsible for its production. Therefore, further research is warranted to explore the source of IL-4 after infection. IL-4 promotes the type 2 immune response, which is detrimental to host protection during infection in mice (13–17). IL-4 can induce the polarization of macrophages toward the M2 phenotype, which is permissive to the proliferation and dissemination of *C. neoformans* and promotes infection of the host by *C. neoformans* (34–38). This appears to contradict the finding that LincR-PPP2R5C deficiency causes an increase in IL-4 but a decrease in pulmonary *C. neoformans* infection. Consistent with reports in the literature, IL-4 neutralizing antibodies significantly reduced the pulmonary fungal burden in WT mice, suggesting that IL-4 is detrimental to the host. However, the pulmonary fungal burden of the LincR-PPP2R5C KO mice treated with the anti-IL-4 neutralizing antibody did not decrease and remained at a level similar to that of the isotype antibody control group. In addition, we detected significantly increased expression of Arg1 in lung macrophages from LincR-PPP2R5C KO mice after infection, suggesting that pulmonary macrophages were polarized toward the M2 phenotype, which can be explained by increased IL-4 levels in the lung tissue of LincR-PPP2R5C KO mice. These results suggest that there may be a mechanism by which LincR-PPP2R5C deficiency can kill *C. neoformans* in the presence of elevated IL-4.

Innate immune cells participate in the first-line defense against pathogens after infection (4), and macrophages and neutrophils have been reported to be involved in the killing of *C. neoformans* (5, 39). We first focused on CD11b^−^CD11c^+^SiglecF^+^ alveolar macrophages and CD11b^+^SiglecF^−^ interstitial macrophages with bactericidal functions (26, 40). Regrettably, compared to those in WT mice, the number and proportion of CD11b^−^CD11c^+^SiglecF^+^ alveolar macrophages in LincR-PPP2R5C KO mice were significantly lower, while no significant differences were detected in the CD11b^+^SiglecF^−^ interstitial macrophages or the subsequent MHC-II^+^ and MH-II^−^ subsets. IL-4 reportedly promotes the maturation and killing of neutrophils (27). Therefore, we tested the fungicidal activity of IL-4 in WT and LincR-PPP2R5C KO neutrophils stimulated with or without IL-4. We found that, compared with WT neutrophils, LincR-PPP2R5C KO neutrophils treated with IL-4 exhibited increased fungicidal ability. Overall, LincR-PPP2R5C deficiency leads to enhanced fungicidal capacity of neutrophils after *C. neoformans* infection, which is associated with elevated IL-4.

NE is the main protease with microbicidal activity in primary neutrophil granules (28–30), and ROS play an important role in host defense against microbial pathogens (31). To further explore the underlying mechanisms involved, we examined the production of NE and ROS, and the results confirmed that the fungicidal activity of neutrophils in LincR-PPP2R5C KO mice depends on NE and ROS in response to IL-4 stimulation. Moreover, *in vivo* experiments verified that the number of neutrophils and the level of elastase in the BALF of LincR-PPP2R5C KO mice were significantly increased. Although the exact mechanism by which LincR-PPP2R5C enhances the fungicidal activity of neutrophils is unclear, the increase in NE and ROS in neutrophils from IL-4-induced LincR-PPP2R5C KO mice may partly explain this finding.

PP2A is the most abundant serine threonine phosphatase in mammals (41). Research has indicated that its activity diminishes in the context of asthma, and it contributes to pulmonary protection through anti-inflammatory mechanisms (42). Moreover, PP2A is involved in various important aspects of cellular function and is related to the innate immune response during infection. To further explore the underlying mechanisms, we measured the levels of total PP2A, phosphorylated PP2A, and methylated PP2A in LincR-PPP2R5C KO neutrophils. The results indicated that LincR-PPP2R5C deficiency, combined with IL-4, increased the level of phosphorylated PP2A in neutrophils. Additionally, the role of PP2A in the production of elastase in LincR-PPP2R5C KO neutrophils was further confirmed by the exogenous addition of a PP2A inhibitor. Overall, LincR-PPP2R5C deficiency results in elevated phosphorylation of PP2A, thereby enhancing the fungicidal effect of neutrophils on *C. neoformans*. This effect is mediated by the production of elastase and ROS, which is facilitated by IL-4.

The results of *in vivo* experiments indicated that LincR-PPP2R5C deficiency led to an increase in the expression of IL-4 in the lung tissue of mice 21 days post infection, along with an increase in neutrophil infiltration in BALF and lung tissue and an increase in elastase content. This finding suggested that in infected mice, the absence of LincR-PPP2R5C enhances the fungicidal capacity of neutrophils, which may be linked to elevated IL-4. However, in reverse validation, the depletion of neutrophils in LincR-PPP2R5C KO mice did not exacerbate *C. neoformans* infection, which may be due to compensatory mechanisms in the body.

There are several limitations in this study. First, although this study demonstrated that IL-4 promoted the killing of *C. neoformans* by neutrophils, which is dependent on LincR-PPP2R5C deficiency, we did not further explore the baseline difference between neutrophils from LincR-PPP2R5C KO mice and those from WT mice, nor did we further study the mechanisms underlying the increase in IL-4 caused by LincR-PPP2R5C deficiency. In addition, LincR-PPP2R5C deficiency impaired IL-4 production in a murine model of allergic asthma (12). The present study revealed that LincR-PPP2R5C deficiency increased IL-4 production in pulmonary cryptococcosis. The regulatory role of LincR-PPP2R5C in IL-4 production warrants further study.

In conclusion, we demonstrate that LincR-PPP2R5C deficiency is protective in pulmonary cryptococcosis, which may be linked with the increased fungicidal activity of neutrophils by increasing the phosphorylation level of PP2A. IL-4 enhances the production of elastase and ROS in LincR-PPP2R5C KO neutrophils, which may partially explain the paradoxical roles of IL-4 in pulmonary cryptococcosis.

## Data Availability

The data sets used and/or analyzed in the study are available from the corresponding author upon reasonable request.

## SUPPLEMENTAL MATERIAL

Fig. S1 mbio.02130-24-s0001.tif The increased IL-4 in LincR-PPP2R5C KO mice after infection was mainly derived from non-T cells.

Fig. S2 mbio.02130-24-s0002.tif CD4^+^ cell depletion did not impact the expression level of IL-4 in the lung tissue of infected mice.

Fig. S3 mbio.02130-24-s0003.tif L-4 failed to augment *C. neoformans* killing by LincR-PPP2R5C KO macrophages *in vitro*.

Fig. S4 mbio.02130-24-s0004.tif Neutrophil depletion *in vivo* did not alleviate pulmonary cryptococcosis.

Legends mbio.02130-24-s0005.docx Supplemental figure legends.

ASM does not own the copyrights to Supplemental Material that may be linked to, or accessed through, an article. The authors have granted ASM a non-exclusive, world-wide license to publish the Supplemental Material files. Please contact the corresponding author directly for reuse.
