# Supplementary figures and images for: LincR-PPP2R5C deficiency enhancing the fungicidal activity of neutrophils in pulmonary cryptococcosis is linked to the upregulation of IL-4
Source: mBio. 2024 Sep 17;15(10):e02130-24. doi: 10.1128/mbio.02130-24 (PMC11481880; doi:10.1128/mbio.02130-24)

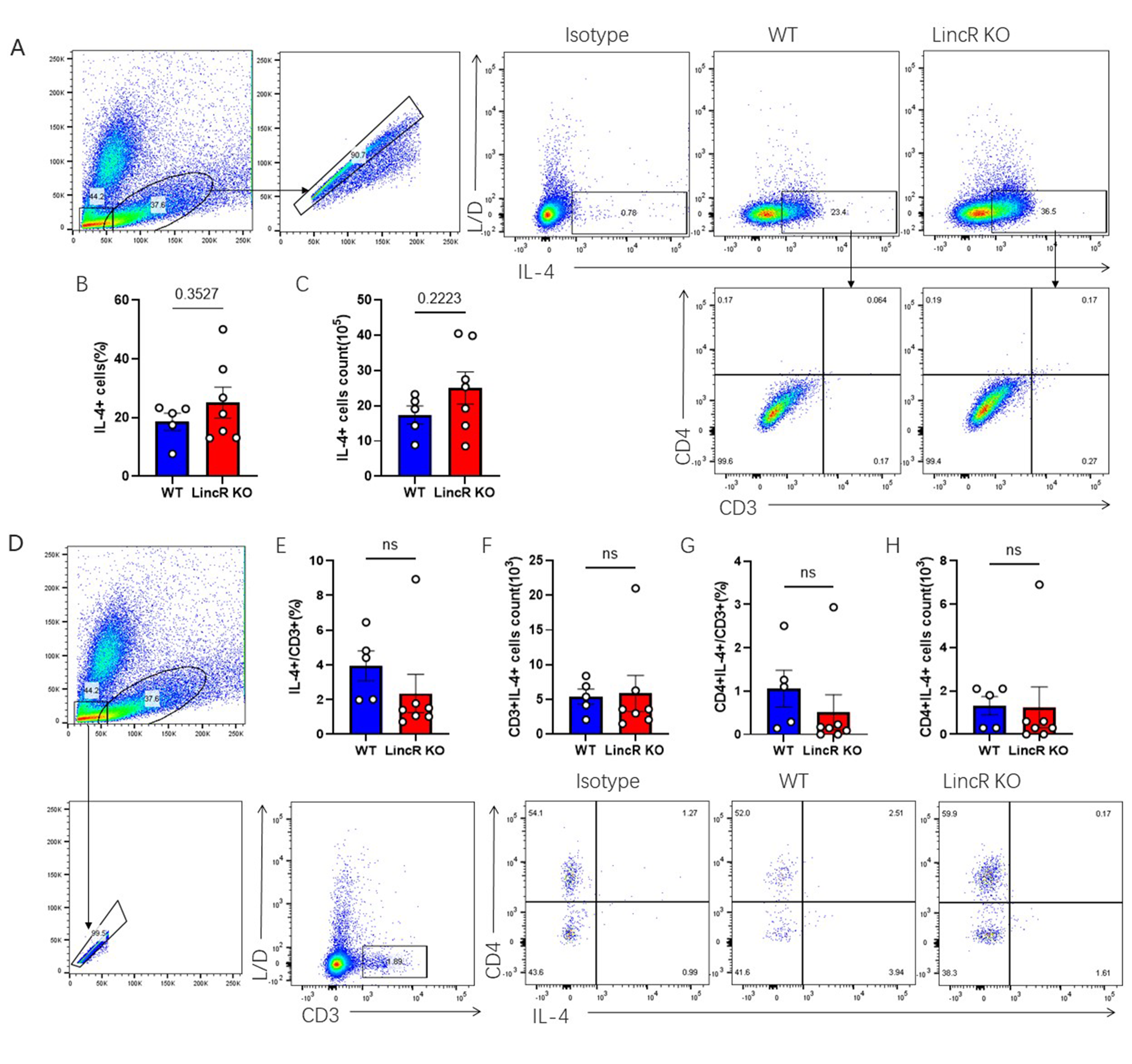

Supplement: Fig. S1 — The increased IL-4 in LincR-PPP2R5C KO mice after infection was mainly derived from non-T cells. [file mbio.02130-24-s0001.tif]

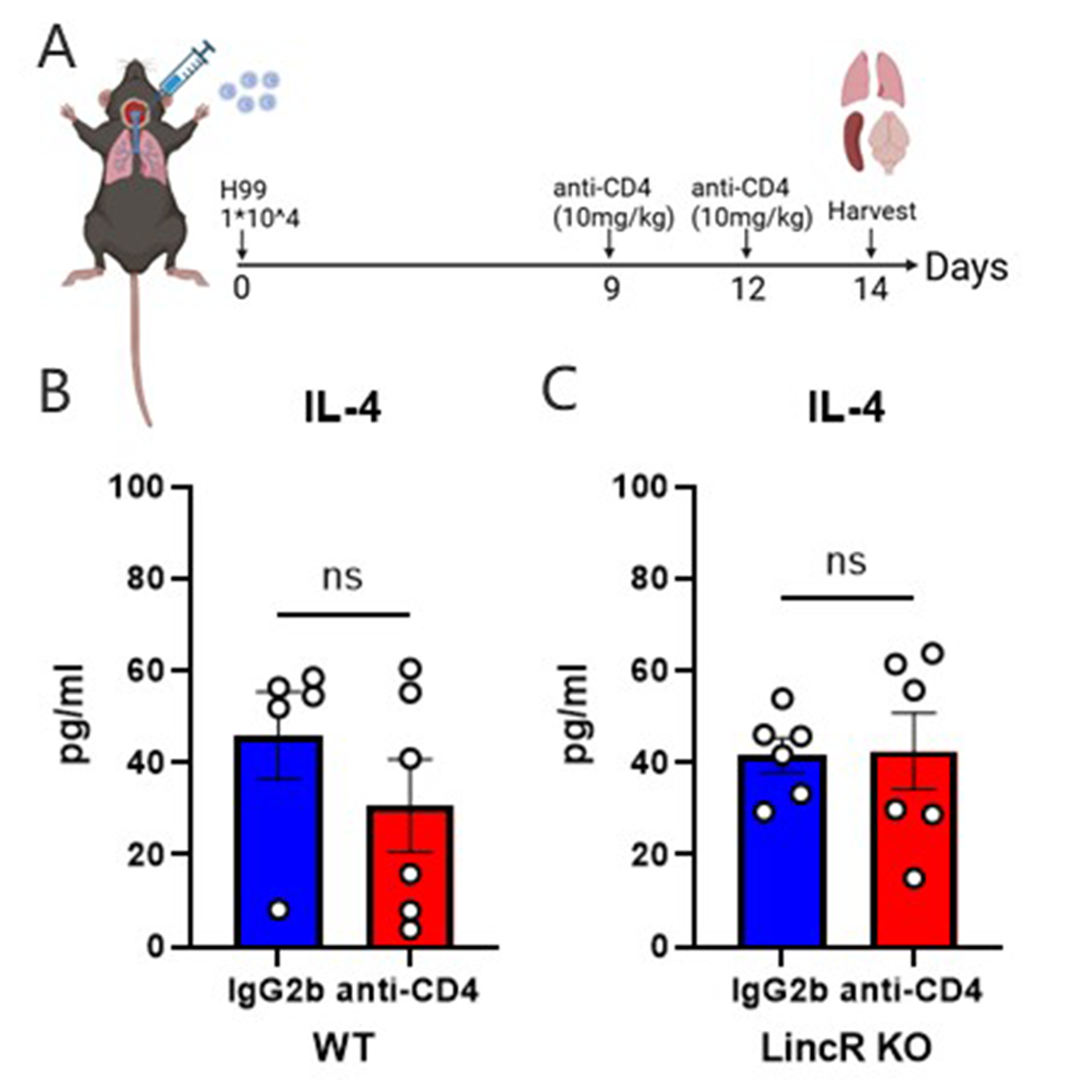

Supplement: Fig. S2 — CD4+ cell depletion did not impact the expression level of IL-4 in the lung tissue of infected mice. [file mbio.02130-24-s0002.tif]

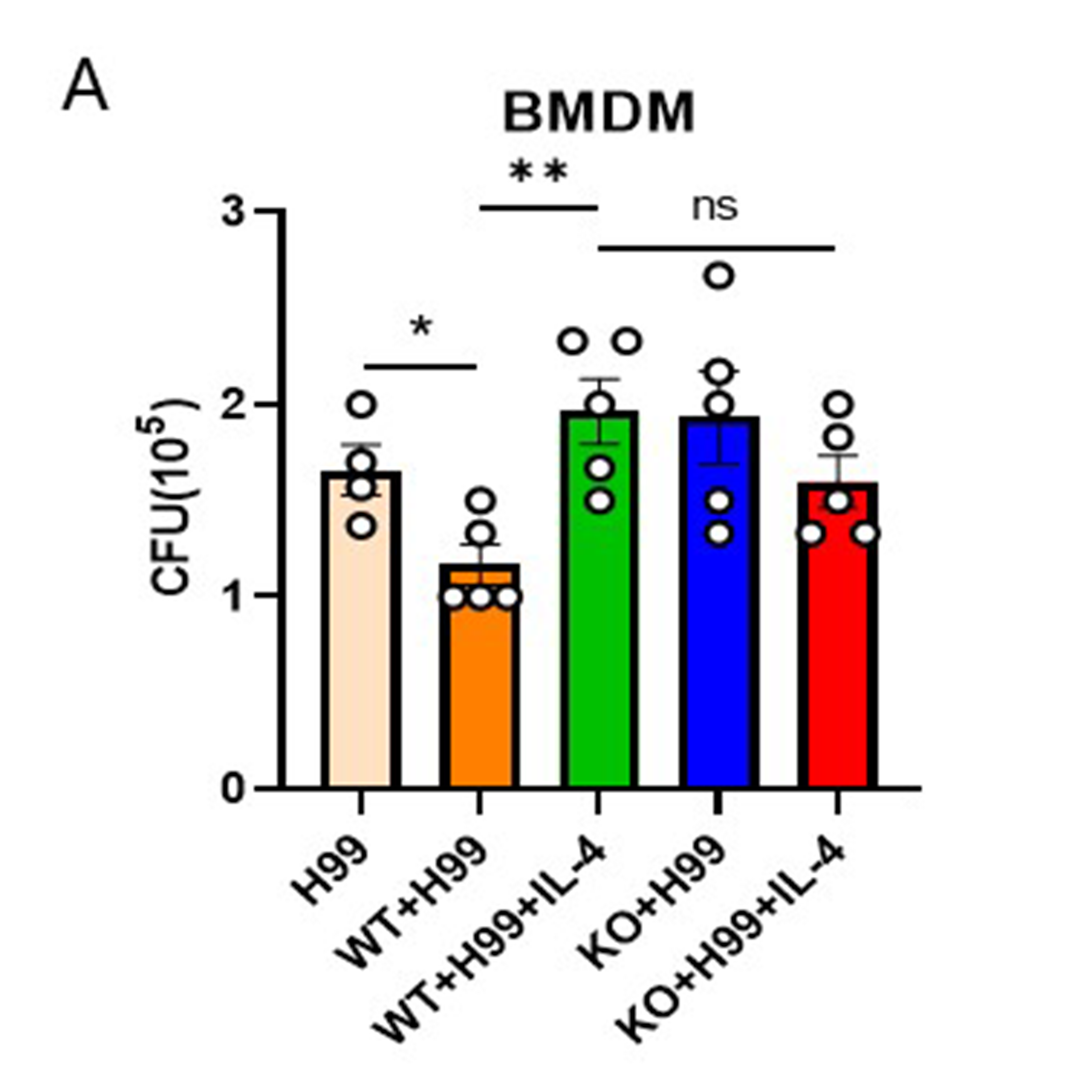

Supplement: Fig. S3 — L-4 failed to augment C. neoformans killing by LincR-PPP2R5C KO macrophages in vitro. [file mbio.02130-24-s0003.tif]

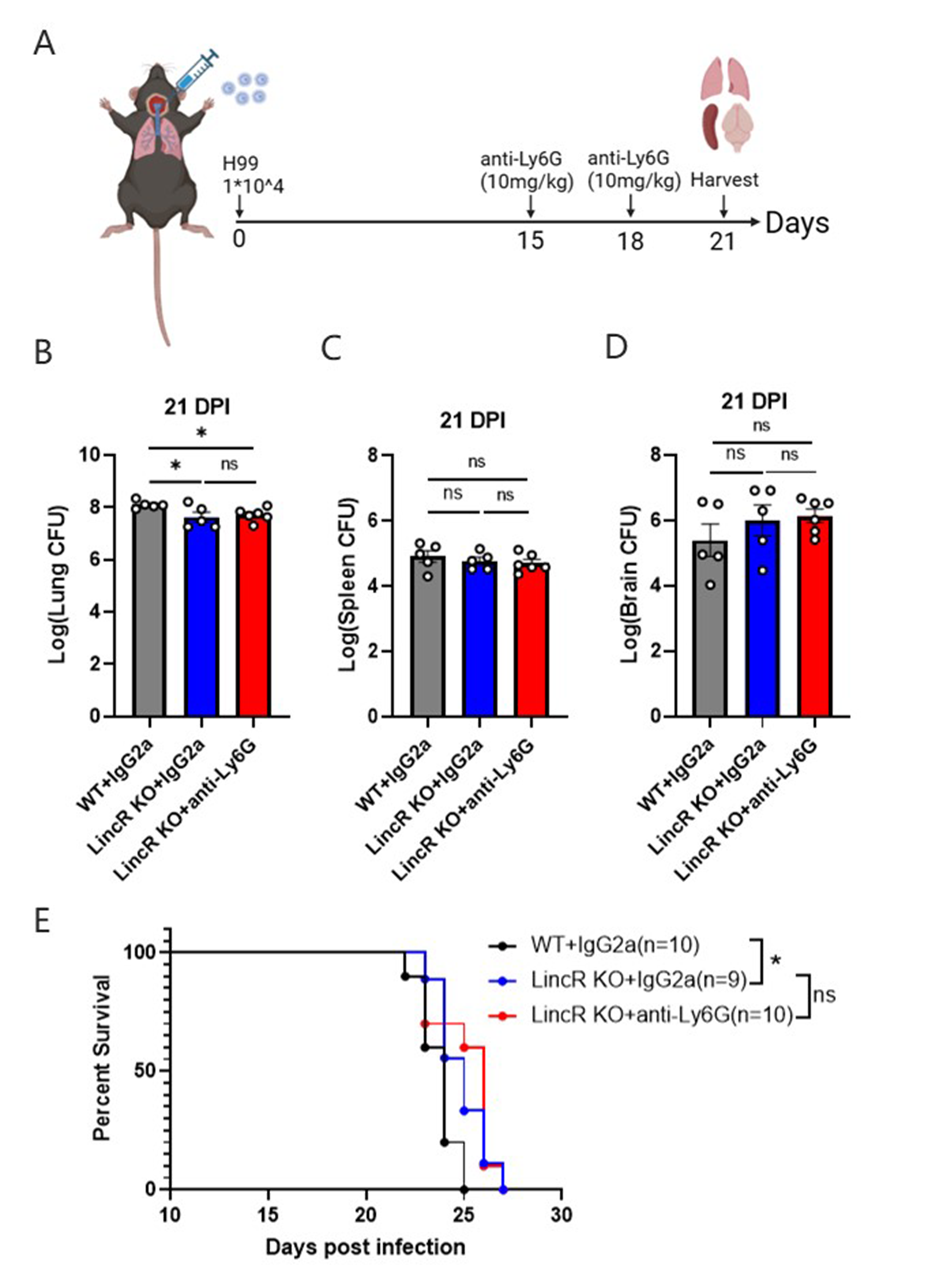

Supplement: Fig. S4 — Neutrophil depletion in vivo did not alleviate pulmonary cryptococcosis. [file mbio.02130-24-s0004.tif]
