# Supplementary material for: LincR-PPP2R5C deficiency enhancing the fungicidal activity of neutrophils in pulmonary cryptococcosis is linked to the upregulation of IL-4
Source: mBio. 2024 Sep 17;15(10):e02130-24. doi: 10.1128/mbio.02130-24 (PMC11481880; doi:10.1128/mbio.02130-24)
Supplement: Legends — Supplemental figure legends. [file mbio.02130-24-s0005.docx]

**Supplementary Figures**

**Figure** **S1 The increased IL-4 in LincR-PPP2R5C KO mice after infection was mainly derived from non-T cells.** WT and LincR-PPP2R5C KO mice were infected intratracheally with 1 × 10^4^ CFU of *C. neoformans* strain H99 and sacrificed at 21 days postinfection. Total lung cells were analyzed by flow cytometry. (A) Gating strategy for IL-4^+^ cells. (B) Percentage of live IL-4^+^ cells. (C) Number of IL-4^+^ cells. (D) Gating strategy for lymphocytes. (E) Percentage of IL-4^+^ cells among CD3^+^ cells. (F) Number of CD3^+^IL-4^+^ cells. (G) Percentage of CD4^+^IL-4^+^ cells among CD3^+^ cells. (H) Number of CD4^+^IL-4^+^ cells. The data are shown as the mean ± SEM (n=5-7 mice per group). **P* < 0.05; ns, not significant; unpaired Student’s t test.

**Figure** **S2 Elimination of CD4^+^ cells did not impact the expression level of IL-4 in the lung tissue of infected mice.** WT and LincR-PPP2R5C KO mice were challenged intratracheally with 1 × 10^4^ CFU of *C. neoformans* strain H99 and were given CD4 antibody or the IgG2b isotype control, respectively, at 7 days postinfection (200 μg per mouse), twice a week. The mice were then sacrificed at 14 days postinfection. (A) Schematic of drug administration for mice infected with *C. neoformans*. (B) The expression level of IL-4 in the lung tissue of WT mice. (C) The expression level of IL-4 in the lung tissue of LincR-PPP2R5C KO mice. The data are shown as the mean ± SEM (n=5–6 mice per group). **P* < 0.05; ***P* < 0.01; ns, not significant; unpaired Student’s t test.

**Figure S3 IL-4 failed to augment *C. neoformans* killing by LincR-PPP2R5C KO macrophages in vitro.** (A) Fungicidal activity of WT and LincR-PPP2R5C KO bone marrow-derived macrophages against *C. neoformans* stimulated with or without IL-4 (15 ng/ml) (n=4-5 per group). The data are shown as the mean ± SEM. **P* < 0.05; ***P* < 0.01; ns, not significant; unpaired Student’s t test.

**Figure S4 Neutrophil depletion in vivo did not alleviate pulmonary cryptococcosis.** WT and LincR-PPP2R5C KO mice were challenged intratracheally with 1 × 10^4^ CFU of *C. neoformans* strain H99 and were given Ly6G antibody or the IgG2a isotype control at 14 days postinfection (200 μg per mouse), twice a week. The mice were then sacrificed at 21 days post infection. (A) Schematic of drug administration for mice infected with *C. neoformans*. (B-D) CFUs in the lungs, spleens and brains of infected mice. **P* < 0.05; ns, not significant; unpaired Student’s t test. (E) The survival of WT and LincR-PPP2R5C KO mice was monitored daily postinfection. **P* < 0.05; ns, not significant, using a log-rank test.
